# The Royal London Ophthalmic Hospital

**Published:** 1892-05-28

**Authors:** 


### THE ROYAL LONDON OPHTHALMIC HOSPITAL.

### £35,000 Wanted for Rebuilding.

A meeting for the purpose of raising funds to rebuild the Royal London Ophthalmic Hospital at Moorfields, which was established at the beginning of this century, was held at the Mansion House on the 20th inst., the Lord Mayor presiding. H.R.H. the Duke of Cambridge, who was to have been present, was prevented, through illness, from attending.

The Lord Mayor gave a brief history of the institution since its foundation, as the "London Dispensary for the Relief of the Poor Afflicted with Eye Disease," in 1805. So much good and useful work did the institution succeed in doing that in 1820 the accommodation then existing was found to be quite inadequate, and in the following year the foundation stone of the present hospital building was laid. In 1869 the hospital had to be again extended by the addition of a new wing, towards which Her Majesty the Queen (who, since 1836, when, as the Princess Victoria, she first became connected with the institution, had taken a great interest in its work) contributed largely. (Applause.) Since the year 1869 the work of the Moorfields hospital had completely outgrown its quarters, and the Board of Management had resolved upon the erection of a new building altogether, which would be suitable, both hygienically and scientifically, to the ever-increasing calls upon its resources. This step would necessitate an expenditure of £35,000, but he did not think it would be so difficult to raise this sum when they recognised that this charity in relieving patients suffering from a defect in their eyesight in many cases actually came between those people and total absolute blindness, which probably meant the loss of the breadwinner to the family. (Applause.)

Sir John Lubbock, Bart., M.P., after expressing the regret they all felt at the absence of the Duke of Cambridge, proposed the following resolution:—

That this meeting desires to express its deep sense of the importance and usefulness of the Royal London Ophthalmic Hospital, Moorfields, which by its beneficent work has, for nearly ninety years, afforded an immense amount of relief to the poorer classes when suffering from diseases of the eye, which, even in their slighter forms, are more or less incapacitating for work, and, on their graver manifestation, lead to blindness with all its attendant calamities. This meeting further records its hearty appreciation of the hospital as a school of ophthalmic medicine and surgery, which, through the instruction given to its students, extends the benefits of skilful treatment to all classes of sufferers, not only in the United Kingdom, but throughout all parts of the world.

People felt very often, when appeals were made for large sums of money for the purpose of improving the social condition of their fellow men, that while such movements might appeal strongly to their sympathy, there was still an element of doubt as to whether in practice they would be carried out as successfully as the promoters would seem to indicate. In the present instance, however, they were not appealing to the public for funds for a new institution, nor for any new object, but for a charity which had been tried, through many years, and had proved its great usefulness. (Applause.) In 1861 the number of in-patients treated was 760, out-patients 15,000, and attendances 71,000. In 1871 the in-patients had risen to 1,000, the out-patients to 18,000, and the attendances to 95,000. In 1881 the in-patients were 1,700, out-patients 23,000, and attendances 104,000; while in 1891 the figures were respectively 1,900, 25,000, and 110,000. Thus they would see there had been a great and growing increase in the numbers of persons attending the hospital, which had at length become really more than could be dealt with, the result being the turning away of many who, with every justification, came to it for relief. They were often obliged to turn the in-patients out of the hospital earlier than was desirable in order to take in other serious cases which would not brook delay, and the number of out-patients that attended made the accommodation very scanty and inconvenient. The Royal London Ophthalmic Hospital had, therefore, a very good case on which to base an appeal for funds. The medical profession, amongst whom he made careful enquiries when he became President of the hospital, regarded it as a model institution, which was known throughout Europe, he might almost say throughout the civilised world, as being perhaps the very best of its kind in existence. (Applause.)

Sir Reginald Hanson, Bart., M.P., seconded, saying that the Royal London Ophthalmic Hospital had most pre-eminently a claim to the support of the Mansion House, and through the Mansion House of the support of the citizens of London.

The resolution was carried unanimously.

Mr. Jonathan Hutchinson (late President of the Royal College of Surgeons), who said he had been connected with the hospital since his student days, proposed the following resolution :—

That it is obviously of the very highest importance that the medical and surgical work of the Royal London Ophthalmic Hospital, Moorfields, should be conducted under the most favourable conditions which modern science and experience can suggest, and, in view of the utter inadequacy of the present building to comply with the advanced rules of hospital construction, this meeting is of opinion that the hospital should be entirely rebuilt, with provision for a much larger number of in-patients than can now be accommodated, and cordially recommends that an appeal be made to the public for the sum of £35,000 for this purpose, pledging itself to use every effort to assist the Committee of Management of the hospital in their endeavour to raise this amount.

Mr. Hutchinson said that not one single word uttered by the previous speakers, not one single word which had been placed in either resolution, was in the slightest degree an exaggeration. (Hear, hear.) His memory of the institution went back to the time when it was in a very much humbler condition than it was at present. He remembered quite well how, when they took possession of the rooms they at present occupied, the medical staff had thought them simply palatial. Now, however, the hospital had utterly outgrown its present buildings, this state of affairs having been brought about by the immense strides which ophthalmic science had made of late years. As a school of ophthalmic surgery the Royal London Ophthalmic Hospital was not a hospital for London only, nor even for England alone, but it was an institution the usefulness of which was spread over all parts of the English-speaking world—he might go farther and say over all parts of the civilised world, for students from all parts of Europe and America came there to study. (Applause.)

Mr. Burdett seconded the resolution, which was carried unanimously. Mr. Burdett said that in order to make good its claim to so large a sum as £35,000 it was necessary that the hospital should show that it fulfilled three requirements, (1) that the proposed extension was necessary, (2) that the money would be well spent, and (3) that an adequate income to maintain the institution would be forthcoming when the new building was erected. That the first requirement was fulfilled those who knew the work of the Moorfields hospital could have no doubt, while a visit to the hospital would speedily convince those who did not of the utter inadequacy of the present buildings. As to the second, the very terms of the resolution were some guarantee that the money would be well spent, while a further guarantee of this was the fact that they had selected as architect Mr. Keith D. Young, a gentleman whose work he was familiar with, and one who had this signal merit, that he never exceeded his estimates. (Hear, hear.) For the third requirement they had in reserve a mine which he hoped the Committee would work. No class of case was, on the whole, so well able to pay something for treatment as the class who attended the ophthalmic hospitals. He therefore hoped, as he saw that at present no such item as "patients' payments" appeared in the accounts, that when the new building was erected arrangements would be made to afford those patients who might be able and willing to give something for their treatment, a chance of so doing. These three requirements being fulfilled, the question arose, who was to give the money? The institution, besides being of European, or rather of international fame, was more than all a national hospital in that it ministered to the needs not only of Londoners but of the poor who were suffering from ophthalmic diseases in all parts of the United Kingdom. They had, therefore, not only the right to go to London for the money, but they had the right to call upon the provinces as well. His own view was that if the amount required was to be roughly divided into £20,000 from London, and £15,000 from the provinces, that would be a fair division, and in regard to the provincial contribution, if the Committee communicated with the mayors of the thirty largest cities and ask for £500 from each they would find that that money would be readily forthcoming. (Applause.)

Among the subscriptions which have already been received the surgical staff of the hospital have promised £1,000; the Goldsmiths' Company, £500; the Corporation of London and the Treasurer (Mr. J. Deacon), 200 guineas each; and the Merchant Taylors' Company and Sir John Lubbock (the President), 100 guineas each.

